# Medicalizing disabled people's emotions—Symptom of a dis/ableist society

**DOI:** 10.3389/fsoc.2023.1230361

**Published:** 2023-12-11

**Authors:** Yvonne Wechuli

**Affiliations:** ^1^Section Disability, Inclusion, and Social Participation, Department of Social Work, Faculty of Human Sciences, University of Kassel, Kassel, Germany; ^2^Section Theory of Special Education and Rehabilitation, Department Special Education and Rehabilitation, Faculty of Human Sciences, University of Cologne, Cologne, Germany

**Keywords:** disability, dis/ableism, emotion, medicalization, compulsory able-mindedness, impairment effects, hysteria, fetishization

## Abstract

The theoretical–conceptual article at hand explores how emotional discourses shape social relations by specifically focusing on the medicalization of disabled— and chronically ill—people's emotions. Medicalization is a concept from medical sociology that describes medicine's expansion into non-medical life areas, for instance into the realm of emotions, sometimes in order to challenge this expansion. The emotions of disabled people are often presented as a medicalized problem, rather than recognizing their embeddedness in a dis/ableist socio-cultural context. Such discourses instrumentalize feelings in order to individualize the responsibility for disability. For a contextualized and emancipatory approach, this study reviews papers on medicalized emotions from Disability Studies—a research program that can provide a rich archive of experiential accounts yet to be theorized through a comprehensive emotional perspective. The medicalization of disabled people's emotions can manifest in different ways: (1) In a dis/ableist society, able-mindedness is *compulsory*; i.e., we fail to question that a healthy mind is the norm and something to strive for unconditionally. This is also true on an emotional level; after all, some medical diagnoses are based on the *wrong* degree or temporality of emotionality. (2) Unpleasant feelings such as sadness are misunderstood as symptoms of *impairment* rather than effects of discrimination. (3) The expression of hurt feelings, e.g., related to discrimination, can easily be dismissed as *hysterical*. This assumption epistemologically disables patients. (4) Love and desire are delegitimized as *fetish*, for example, the desire for a disabled lover or the wish to start a family despite a chronic illness. The medicalization of disabled people's emotions individualizes and delegitimizes unpleasant emotions that emerge in a dis/ableist society. Different facets of medicalization enforce medical treatment instead, albeit in different ways. Disabled and sick people are cast as not feeling and desiring the right way, while hegemonic discourse prescribes psychological treatment against the effects of discrimination and bodily symptoms it cannot explain. Beyond the dismissal of disabled people's experience, adverse effects on healthcare delivery and health outcomes can be expected.

## 1 Introduction

The medicalization of emotions is one phenomenon where the sociologies of health and emotion intersect. The concept of medicalization was coined by conflict theorists in medical sociology to describe the expansion of medicine's mandate into ever more areas of life—including the management of emotions—and, at times, to challenge this expansion (Thomas, 2009, p. 30–31).

Challenging the medicalization of *disability* constitutes a founding idea of the field of Disability Studies^1^ (Thomas, 2009) that this article draws on.

“Medicine sustains and enhances lives. Our concern lies more with *medicalisation*: a persuasion that shoe-horns all perspectives into a grand narrative dominated by the vocabularies of medicine. Medicalisation refers to the institutionalization of medical knowledge as *the* knowledge through which to make sense of human diversity and deviations from human norms” (Goodley et al., 2022, p. 18).

Disability Studies criticize the medicalization of disability as individualizing disability, instead of acknowledging it as a *social* problem. They have accused particularly medical sociology of perpetuating an individualizing *tragedy narrative* of disability through a focus on impairment and limitation (Thomas, 2021).

Within the sociology of emotion, medicalization has been discussed as transforming emotional deviance into a medical problem in order to expand medicine's jurisdiction (Payton and Thoits, 2011). While not feeling according to situational expectations is a common phenomenon, clinicians and laypeople alike categorize many mental disorders by deviant emotionality, that is, a violation of emotional norms (Thoits, 2012). “Emotional deviance refers to persistent, repeated, or intense violations of societal feeling or expression norms, where emotion management efforts are often ineffective” (Thoits, 2012, p. 201).

The medicalization of emotional deviance as mental disorders may be beneficial to the subject whose experience and behavior are labeled as such because it withdraws individual blame for deviant emotionality (Payton and Thoits, 2011) and provides access to medical treatment (Thoits, 1990; Payton and Thoits, 2011). However, a medicalization of emotion individualizes and depoliticizes a social problem—in this case, emotions that cause suffering (Payton and Thoits, 2011), while it may entail involuntary treatment (Thoits, 1990). Moreover, emotional norms vary across socio-cultural groups and individual beliefs, such that similar emotional displays are judged (or, even, diagnosed) differently (Thoits, 2012) and a deviant emotionality is distributed differently across social groups (Thoits, 1990).

This study particularly focuses on the medicalization of disabled people's emotions—arguing that their subjective experiences of illness are dismissed as a pathology to be treated (Johnson, 2011). Moreover, it is necessary to pay attention to the emotional consequences of living in a dis/ableist society, so that these are not misunderstood as quasi-natural consequences of impairment (Thomas, 1999). After all, Disability Studies have long been called out to engage with conscious and unconscious subjective disability experiences to prevent “a theoretical vacuum is left, which is filled by those who adopt an individualistic and decontextualized perspective” (Marks, 1999, p. 611).

As I have argued elsewhere (Wechuli, 2022), Disability Studies can offer the sociology of emotions a vast archive of emotional first-hand accounts explored from a subaltern and emancipatory perspective but not yet comprehensively theorized through an emotional lens. This archive comprises accounts of a vast array of different impairments and illnesses, e.g., amputated limbs, autism, cancer, chronic pain, or fatigue. Some authors draw on first-person experiences but do not make their medical diagnosis explicit, while others generalize their observations. Furthermore, it seems likely that the socio-cultural function of emotions in sustaining, experiencing, or struggling against discrimination based on other markers of difference might be similar.

The article at hand will explore how disabled people's emotions are *dismissed* by medicalization. This dismissal works in different ways. Some emotions are pathologized *per se* as *able-mindedness* is compulsory (see Section 3.1). Unpleasant emotions like grief or even depression tend to be not only pathologized but also misread as *impairment effects* (Thomas, 1999, p. 43) rather than effects of dis/ableism (see Section 3.2). Expressing distress or pain that cannot be explained by hegemonic medical knowledge easily leads to the accusation of *hysteria*—of intentionally falling sick for secondary *gain* (see Section 3.3). Furthermore, desiring to start a family despite a chronic health condition or even desiring a disabled partner is delegitimized to be a *fetish* (see Section 3.4). To start, the medicalization of disabled people's emotions is contextualized in a dis/ableist society (see Section 2).

## 2 Dis/ableist society

The article at hand draws on a PhD project that seeks to outline the theorization of emotion, affect, and feeling as socio-cultural phenomena with material consequences in order to find a (still pending) common language for addressing emotional issues in Disability Studies. To this end, I scanned all Disability Studies journals since their inaugural volumes—applying a wide definition of Disability Studies that encompasses all social and cultural studies research on disability rather than only activist research connected to disability rights movements (Waldschmidt, 2020). I excerpted all papers with emotion-related topics of sociological or Cultural Studies interest and inductively developed a taxonomy to which I assigned the identified articles. Excerpts, taxonomy, and assignments were continuously revised^2^.

Namely, I argue to distinguish: (1) Repertoires of emotion (von Poser et al., 2019) in the dis/ableist imaginary in the sense of socio-culturally acquired *re*actions^3^ to disability from (2) the disabling impact of these emotional *re*actions on disabled people's lives and (3) feeling strategies promoted by disabled activists and scholars to navigate dis/ableism's emotional toll (Wechuli, 2022).

I discuss the medicalization of disabled people's emotions as one disabling impact of socio-culturally acquired *re*actions to disability in the dis/ableist imaginary. The neologism dis/ableism foregrounds that disablism and ableism “can only ever be understood simultaneously in relation to one another” (Goodley, 2014: xiii) as socio-culturally co-constructed notions. “Not all of us experience disablism. We are all plunged into the mire of ableism” (Goodley, 2014, p. 37). In her relational definition of disability, the medical sociologist Thomas (1999, 2009) argued to distinguish^4^ disablism from immediate effects of (physical, sensory, mental, cognitive) impairment.

“Put at its simplest, impairment effects refer to those restrictions of bodily activity and behavior that are *directly attributable* to bodily variations designated ‘impairments' rather than to those *imposed upon* people *because* they have designated impairments (disablism)” (Thomas, 2009, p. 136).

Ableism naturalizes an alleged standard of the bodymind as typical for the human species (Campbell, 2009). An ableist logic contains information on what is considered *normal*—namely, an able body and mind that one should strive for, and divides people into allegedly distinct categories of either *normal* or *not normal* (Campbell, 2019). A dis/ableist imaginary, thus, consists of disablist and ableist perceptions of self and others, which are acquired in a specific socio-cultural context.

Importantly, theory building of Disability Studies on emotional *re*actions to disability mainly relies on reconstructive sense-making. Disabled scholars ruminate about the emotional foundation of discriminatory behavior they (or their disabled study participants) are experiencing (Thomas, 1999; Watermeyer, 2009; Saerberg, 2011; Schönwiese, 2011; Kafer, 2012; Reeve, 2015; Hutson, 2016; Sheppard, 2019)—drawing on sociological, philosophical, anthropological, and psychoanalytical theories rather than empirical data from first-person perspectives. This is different from theory building of Disability Studies on emotional impacts on disabled people's lives and feeling strategies to navigate dis/ableism's emotional toll. Both centrally draw on (auto)biographical and (auto)ethnographic data that provide more depth to emotional episodes—following the Disability Studies principle to foreground disabled people's lived experiences.

Thus, the subjective experience of different emotions triggered by disability remains somewhat speculative. Nonetheless, socio-culturally shaped emotional *re*actions to disability impact on disabled people's lives. I termed these impacts *disabling affect* to stress two aspects:

1. I argue to talk of *affect* (in the singular) as “*relational dynamics*” (Slaby and Mühlhoff, 2019, p. 27; their emphasis) that—in distinction to emotion concepts—cannot be fully captured in words (Slaby and Mühlhoff, 2019). In a spinozist–deleuzian understanding, affect delineates a (1) relational ontology that does not allow us to neatly distinguish body from mind, (2) the inseparability of active involvement (affecting) and receptive involvement (being affected), (3) a capacity inextricably linked to power (Slaby and Mühlhoff, 2019). To discuss the impact of emotional *re*actions to disability on disabled people's lives, it is not necessary to distinctly name the triggered emotion first. Still, the effects of a fearful reaction to disability will differ from effects of a hostile reaction or effects of a disgusted reaction (Nussbaum, 2013; Ahmed, 2014) as “naming emotions involves different orientations toward the objects they construct” (Ahmed, 2014, p. 14). In the current moment in theory building, where we cannot be sure about (named) emotions triggered by disability, we can still listen to disabled people regarding the question of what affective *re*actions to disability *do*.
2. The focus on the impacts of affect is captured by the term *disabling*. *Disabling* hints at the “‘weightiness' of feelings, the way in which feelings are, in some sense, material, such as objects, feelings do things, and they affect what they come into contact with” (Ahmed, 2014, p. 85). In *Contours of Ableism*, Campbell (2009, p. 166) speaks of the “enormous weight” of dis/ableist imaginaries on disabled people's subjective experience. Several authors claim that Disability Studies should analyze the effects of affect to challenge dis/ableism (Narduzzi, 2013; Cheyne, 2016; Soldatic and Morgan, 2017). The ing-form is, further, supposed to indicate the focus on what emotions *do*, rather than what they *are* (Ahmed, 2014). Compatible with such an understanding of doing emotions is the notion of psycho-emotional disablism as one form of disablism introduced by Thomas (1999, 2009). in shaping how disabled people feel and think about themselves, “psycho-emotional disablism places limits on who they *can be* by shaping individuals' ‘inner worlds', sense of ‘self' and social behaviors” (Thomas, 2009, p. 72). Disability Studies owes much to Carol Thomas for bringing subjective experience back to attention in theorizing (Goodley, 2009) and for defending this theoretical decision against backlash (see e.g., Thomas, 1999, p. 73–75). However, the notion of psycho-emotional disablism also has limitations. After all, this notion is supposed to contribute to the theorization of disability, rather than to a theorization of emotions or affect, namely, a *relational* definition of disability (Thomas, 1999).

To specify what affectivity does in dis/ableism, different processes can be outlined of how affect becomes disabling. Informed by repertoires of emotion in the dis/ableist imaginary, disabled people face violent and material consequences as their lives, integrity, belonging, and livelihoods are threatened. Furthermore, they have to relentlessly perform emotion work (Hochschild, 2012) to comply with prescriptions of how (not) to feel (Wechuli, 2023a). Finally, their own feelings are subject to medicalization as these are instrumentalized to disable them. The latter aspect is the focus of this paper.

## 3 Medicalizing disabled people's emotions

Disabled people's emotions (and sometimes their families', lovers', and friends') have often been framed as a problem, specifically as an individual problem rooted in impairment that requires medical attention. Such discourses instrumentalize emotions to medicalize disability.

The following sections will explore how disabled people's emotions are medicalized in different ways. First, the notion of *compulsory able-mindedness* is introduced to point out that some diagnostic categories are based on the *wrong* amount or temporality of emotions. Second, unpleasant emotions as effects of dis/ableism are often misunderstood as *direct effects of impairment*. Third, illness experiences that hegemonic medical knowledge lacks an explanation for tend to be framed as *hysteria* or intentional illness. Finally, the desire for disabled or chronically ill bodies risks being pathologized as *fetish*.

### 3.1 Compulsory able-mindedness

Drawing on Rich's (1980) notion of *compulsory heterosexuality*, authors of Disability Studies have coined the term compulsory able-mindedness (Kafer, 2013) to convey that able-mindedness is assumed until contrary information is shared. This assumption is harmful as it isolates disabled people and blocks their access to accommodations (Kafer, 2003). Davis (2008, p. 218) detects “a general trend toward the medicalization of virtually every emotional and cognitive state.” Compulsory able-mindedness also comprises the *right* amount and temporality of emotionality (Campbell, 2019). The Diagnostic and Statistical Manual of Mental Disorder expresses such normativity as it characterizes certain diagnoses via “*excessive, unusual, inappropriate*” (Thoits, 2012, p. 208; her emphasis) emotional states. Autism is characterized as a lack of emotion, for example, and schizophrenia as excess.

A somewhat *lacking* emotionality is a central characteristic of common understandings and medical classifications of autism (Billington, 2006). Autism is “framed as a medical and clinical problem of affect, a pathology to be treated” (Duffy and Dorner, 2011, p. 204). The so-called *Theory of Mind* (Baron-Cohen, 1997) features autistic people as incapable of empathy understood as intuitively grasping others' intentions and emotions (Duffy and Dorner, 2011). Autistic people are further depicted as lacking spontaneous interaction and emotional reciprocity (Billington, 2006). When empathy is cast as an innate human capability in a linear model of (evolutionary) progress, autistic people's belonging to humanity is questioned (Duffy and Dorner, 2011).

Several Disability Studies scholars have pointed out that the *Theory of Mind* lacks empathy itself (Billington, 2006; Duffy and Dorner, 2011; Milton, 2012). It imagines its audience to be neurotypical^5^ and ignores not only autobiographical accounts of autistic people but their perspectives in general (Duffy and Dorner, 2011). However, first-hand accounts of people who identify as (*high functioning*) autistic^6^ express complex emotional lives and a subjective meaningfulness of emotional issues (Jones et al., 2001). Often, such accounts actually claim an emotional hypersensitivity with strong, sometimes overwhelming or even unbearable feelings (Billington, 2006). Moreover, autistic people challenge environments to be disabling as they elicit feelings of frustration, sadness, anger, and anxiety (Clarke and van Amerom, 2007). They often experience neurotypical people as wildly inaccurate about autistic people's mental states or motives and even as invasive (Milton, 2012). Many behaviors that are read as stereotypically autistic like avoiding eye contact or staring at objects are actually a defense from emotionally overwhelming stimuli and unpleasant experiences (Billington, 2006).

An *excessive* emotionality that impairs reason—or “a disordered economy in which the supply of emotion exceeded the demand” (Johnson, 2011, p. 191)—is one common definition of schizophrenia^7^. An understanding of schizophrenia as excess justified the medical practice of lobotomy that was supposed to dampen the patients' emotional experience by destroying brain tissue. Rather than *curing* schizophrenia, this procedure aimed at rehabilitation of patients into the national economy by making them lose any shame of performing *simple* labor. In this sense, this medical practice proves to be an extreme example of a medicalization of disabled people's emotions (Johnson, 2011).

“For disability scholars, lobotomy offers arguably the limit case of the medicalization of emotion. As emotion is medicalized, old binaries and hierarchies (reason/emotion, weak emotion/strong emotion, negative emotion/positive emotion) are subsumed under the dominant medical binary of normal/pathological, and intense emotion, negative emotion, and, to a certain extent, emotion itself become characterized as impairments to be remedied by medical intervention” (Johnson, 2011, p. 186–87).

Excess is likewise a key characteristic of obsession. Contemporarily diagnosed as obsessive-compulsive disorder, which is classified as an anxiety disorder—thus, as excessive anxiety—obsession has been medicalized only in post-/modern Western societies. Previously and elsewhere, the same thoughts and behaviors would have been interpreted as eccentric or—in a religious context—as *possessed* (Davis, 2008)^8^. An alleged excessive emotionality is also negotiated in the literary genre of youth novels called *Teen-sick Lit*. This genre frames and naturalizes teenagers' emotional repertoire as consisting of sadness, emotional volatility, and excess, which requires rehabilitation to transition into a *healthy*^9^ adulthood (Elman, 2012). “Teen sick-lit has been key to maintaining an image of always-already sad teenagers in diametrical opposition to ‘happy' children and emotionally ‘stable' adults” (Elman, 2012, p. 178).

Besides questions of lack or excess, the *temporality* of feelings can, similarly, be medicalized. The terminology of post-traumatic stress disorder (PTSD) suggests a pathological relationship to temporality: the inability to experience the violent past as over and the present as safe. In contradiction to state-of-the-art understandings of trauma, the notion of PTSD does not convey that trauma is experienced as ongoing and embodied (Rakes, 2019). Freud's (2004) notion of melancholia similarly transports the idea of a pathological temporality of feelings with *healthy* grief envisioned as following a linear timeline. Samuels (2017) recounts being accused by doctors and relatives of grieving too long^10^ for her late mother.

“When I fell ill just 2 years later, both doctors and relatives wanted to believe it was the result of my stored-up grief, my refusal to stop mourning my mother and move on with my life” (Samuels, 2017, n.p.).

There is also a *right* temporality of how to feel toward disability. Medical understandings of disability confront disabled people with stage models of rehabilitation, which promote the idea that one must live through fixed phases of emotionality to *process* disability—from grief to denial to acceptance (Watermeyer, 2009; Douglas et al., 2021). Able-bodyminded parents of disabled children are confronted with similar stages and coping models that assume a chronology of parental emotionality from shock to disappointment about a disabled child's birth (Douglas et al., 2021). When not complying with prescriptions of normative time, “disabled people are pathologized for not feeling the ‘right' sort of loss” (Watermeyer, 2017, p. 153). However, for people born with impairments the notion of loss and compulsory nostalgia for lost able-bodymindedness might not make sense at all (Kafer, 2013).

“This presumption of loss, one that extends even to people who never ‘possessed' what they allegedly ‘lost,' is a symptom of the compulsory able-bodiedness/able-mindedness challenged by disability studies scholars and activists. It illustrates the extent to which the nondisabled body/mind is the default position, as if all bodies/minds are purely abled until something happens to them, as if mind/body variation were not a common occurrence. We are expected to take up nostalgic positions toward our former selves, mourning what we have lost and what can now never be” (Kafer, 2013, p. 43).

What does compulsory able-mindedness do? In general, the idea that emotionality can have a right amount and temporality pathologizes and polices any aberrations from these norms (Donaldson and Prendergast, 2011). However, the attribution of lack, excess, or a *wrong* temporality of feelings has distinct effects.

When people on the autistic spectrum are imagined as unemotional, neurotypical people can deny any complicity in causing them distress (Billington, 2006). For the sake of assimilation, therapeutical or pedagogical interventions for autistic children often attempt “to destroy the defenses which they might have constructed against the immensity of such dangerous feeling and knowing, and upon which their psychological survival might well depend” (Billington, 2006, p. 7). On a more general level, an understanding of autism based on the *Theory of Mind* neglects that definitions of social situations might vary. This assumption naturalizes neurotypical interpretations and relieves neurotypical people from taking responsibility for their own perceptions (Milton, 2012).

An attribution of *excess* rather makes it compulsory to conceal the alleged excess and to pass (Goffman, 1963) as able-minded, which incites emotion work (Hochschild, 2012) and enforces psychiatric treatment. Therefore, dampened emotions^11^ are often not perceived as a relevant side effect of psychiatric treatment, unless impeding productive labor (Johnson, 2011). Similarly, T*een-sick* novels (see above) establish the management of an *excessive* emotionality as a sign of maturity for teenagers to aspire to (Elman, 2012).

Moreover, the urge to treat any perceived lack or excess of emotion trickles down from mental health service settings to subjective experience in everyday culture (Donaldson and Prendergast, 2011). “[S]ustained feelings of sadness prompt consumers to seek medical and pharmaceutical interventions, while sustained feelings of elation might lead consumers to shun them” (Donaldson and Prendergast, 2011, p. 130).

*Temporal* prescriptions transpire in common allegations against disabled people that they have not processed their disability *yet* (Sierck, 2011); they are either cast as *in denial* or as *maladjusted* (Goodley, 2011). Stage and coping models further psychologize parenting and pathologize the relationships of parents with their children. “Through the normative discourses of the psy-professions, it seems there is no possibility of a sane response to the birth of a disabled child” (Douglas et al., 2021, p. 46).

A first facet of the medicalization of disabled people's emotions transpires when compulsory able-mindedness is extended to the realm of feeling. Able-mindedness is naturalized as the norm when a right extent and a right temporality of feeling are assumed that is imagined to be neatly distinguishable from (intellectual) disability and (mental) illness.

### 3.2 Pathologizing emotions as impairment effects

A widely shared experience among disabled people is the pathologizing of their emotions. Dis/ableism can have an intense emotional toll, but when related feelings are expressed, they tend to be mistaken as *impairment effects* (Thomas, 1999, p. 43). Hence, the source of unpleasant feelings is misattributed as grounded in impairment rather than in an exclusionary society (Goodley and Runswick-Cole, 2011; Chandler and Rice, 2013), economic marginalization (Chandler and Rice, 2013), violence (Voronka, 2017; Evans, 2020), or disabling environments (Clarke and van Amerom, 2007; Goodley and Runswick-Cole, 2011; Boyle, 2014; Fish, 2018). Chandler and Rice (2013, p. 238) speak to an exclusionary society and economic marginalization:

“[I]f disabled people are unhappy, it is because we are mourning our unfortunate circumstances and not because we live in an ableist culture or that a majority of disabled people live under the poverty line.”

To pathologize emotions as impairment effects makes sense in a socio-cultural context that reads disease as an existential threat (Hughes, 2012). Sick and disabled people are, therefore, cast as entangled in affect and emotions—as *unreasonable*—unless they are fighting against disease^12^ and subjecting themselves to medical treatment (Waldschmidt, 2012). Waldschmidt (2012) reminds us of the etymological roots of the word *patient*, which translates to misfortune and affect from the Greek *páthos* or as suffering and patiently (waiting) from the Latin *patiens*.

Misattributions of causality become apparent when efforts to mitigate unpleasant feelings seek to overcome the feelings rather than their trigger (Evans, 2020). Medical staff are called out for prescribing disabled people psychiatric treatment against emotional responses to marginalization (Sanmiquel-Molinero and Pujol-Tarrés, 2019). Secondary mental health issues like anxiety or depression are naturalized as sequelae of autism (Clarke and van Amerom, 2007), which will probably lead to medical and therapeutical treatment as well. People with mental health issues experience that their affective responses to violence or to their liminal status are relegated to psychological, psychiatric, or care settings that erase their emotional suffering (Evans, 2020). One treatment approach for (pathologized) anger and aggression in psychiatric *care* is seclusion—as seen in the forensic setting of locked psychiatric wards for women with learning disabilities who have been involved with the criminal justice system in the United Kingdom. Seclusion rooms are supposed to have the therapeutic effect of letting the locked woman calm down^13^ outside the conflict situation while safeguarding her and others (Fish, 2018). In special education settings, different diagnostic labels are used to justify seclusion for children as young as kindergarten age (Erevelles and Minear, 2010).

Several (institutional) environments have been called out as disabling, hence as the underlying cause of pathologized emotions. The above-mentioned locked psychiatric wards for women with learning disabilities fail “to sufficiently recognize that anger, aggression, self-harm, or violence may be the result of an oppressive institutional environment” (Fish, 2018, p. 145). Instead, they even encourage to tacitly endure violence—although such submissive behavior could trigger re-traumatization due to past experiences of violence (Fish and Hatton, 2017). Submissive behavior could, moreover, actually endanger disabled women who are disproportionately targeted in domestic and sexualized violence (Fish and Morgan, 2019). Psychiatric institutions at large are alleged to frame understandable feelings as symptomatic of mental illness (Abrams, 2014; Fish, 2018; Evans, 2020) when putting people in distressing situations, taking all means of expressing themselves, and then interpreting any behavior as a symptom (Abrams, 2014). The pathologizing of *Mad* people's emotions silences structural violence that service users are subjected to in psychiatry (Voronka, 2017). Emotional agitation, for example, is mobilized to label people as *mentally ill*, while it can be grounded in involuntary institutionalization (Evans, 2020). Both (alleged) excess and lack of emotionality can be subject to pathologizing in psychiatry, while the role of professionals' feelings remains overlooked in diagnostics^14^ (Donaldson and Prendergast, 2011) or special education assessment (Erevelles and Minear, 2010).

For people with dementia, aggressive behavior and unpleasant emotions are cast as symptomatic of their neurodegenerative illness. However, institutional care regimes often (unnecessarily) constrain choices that can prompt emotional reactions, for instance, in the form of aggressive behavior. The widespread assumption that people with dementia are increasingly unable to choose or express their preferences and aspirations has to be revised when choice-making is understood as an embodied and socio-emotional process, which can be enabled by assistance (Boyle, 2014). School settings are similarly stressful environments that may have an (even overwhelming) emotional toll on children and their parents. Yet antecedents of (physical) violence in conflicts among students or between students and teachers are often silenced. Students' behavior and actions are judged against a different standard than the teachers'—even as the management or communication of feelings are declared learning objectives. The teachers' emotional needs are prioritized when students are framed as a burden, a source of stress, or even a threat (Goodley and Runswick-Cole, 2011).

What do such misattributions do? To pathologize understandable emotional reactions to dis/ableism as impairment effects invalidates these emotional reactions (Goodley and Runswick-Cole, 2011) and can lead disabled people to blame themselves for their feelings (Sanmiquel-Molinero and Pujol-Tarrés, 2019). When anger is pathologized as an impairment effect, disabled people are framed as particularly dangerous (Fish, 2018). Disabled people have often been cast as violent or even hostile (Goodley and Runswick-Cole, 2011) and diagnosed as showing *challenging* behavior (Goodley, 2017), which potentially legitimizes segregation or even abusive institutional care.

A second facet of the medicalization of disabled people's emotions surfaces when understandable emotional reactions to discrimination and marginalization are misunderstood as direct *impairment effects*. Disabled people are, thus, instructed to change their feelings or even have them treated, rather than to criticize conditions that evoke, e.g., anger in the first place. Such misattributions hint at able-bodyminded people's attempt to distance themself from their own feelings related to their complicity in complex social injustice.

### 3.3 Hysteria

An article on the medicalization of emotions would not be complete without reference to an outdated diagnosis: hysteria. Drawing on Freud's case history of his patient Dora (Freud and Rieff, 2005) case history of his patient Dora, Mollow (2014, p. 191) understands the diagnosis of hysteria as “epistemological disablement.” Hysteria's key characteristic is a body that falls sick willingly due to unconscious motives. This diagnosis—whether assigned literally or implicitly—disables because it cannot be refuted (Atkins and Hodges, 2010; Mollow, 2014). “Ironically, according to the paradigm, the more the patient is adamant that her symptoms are real, the more clinicians presume that they are purely the result of intrapsychic phenomena” (Atkins and Hodges, 2010, p. xxii). The assumption of unconscious motives to fall sick invites speculations over psychological (or even spiritual) causes of illness (Wendell, 1996). “[U]nprovable theories are generated to explain how someone would have avoided becoming ill” (Wendell, 1996, p. 96). Overgeneralizations of psychological causes, at times, even continue once a somatic disease has been established in individual cases (Wendell, 1996; Patsavas, 2023) or for a diagnostic category. Beliefs in the psychological causation of, for instance, cancer linger in the pubic imaginary even though its causes have been established to lie in environmental toxins (Wendell, 1996). People with acquired brain injury unquestioningly acquire their impairments, which often include changes in attention, memory, or fatigue, by physical force. Still, they frequently have to navigate irrelevant advice like thinking positively with regard to such neurologically induced cognitive and emotional changes (Hellem et al., 2018).

To date, hysteria does not serve as an official diagnostic category anymore, yet the underlying ideas prevail. Across a range of different diagnostic labels, sick people are blamed for causing their own impairment for secondary *gain* (Atkins and Hodges, 2010; Mollow, 2014; Patsavas, 2023), e.g., people living with chronic pain (Sheppard, 2018; Patsavas, 2023), people with myalgic encephalomyelitis, respectively, chronic fatigue syndrome (Wendell, 1996), people with long COVID (Bê and Sheppard, 2023), people diagnosed with borderline personality disorder (Johnson, 2015), or people with environmental illness, who intensely react to the exposure to everyday toxic chemicals (Mollow, 2014). Imagined secondary gains include access to disability pensions (Atkins and Hodges, 2010), pain medication, time off work (Sheppard, 2018), and becoming the focus of others' attention (Atkins and Hodges, 2010; Mollow, 2014; Johnson, 2015) or sympathy (Atkins and Hodges, 2010).

“[T]he belief that people in pain remain so because we either do not want to get better or actively benefit from our pain remains strangely persistent across both cultural and professional spheres” (Patsavas, 2023, p. 202).

Clearly, we cannot meaningfully write about the notion of hysteria without acknowledging its entanglement with gender stereotypes (Wendell, 1996; Atkins and Hodges, 2010; Mollow, 2014; Johnson, 2015; Goodley, 2017; Sheppard, 2018; Patsavas, 2023) and racialization (Johnson, 2015; Sheppard, 2018; Patsavas, 2023). As notions of physical and mental health are oriented toward a masculine ideal (disabling) behavior is expected of women at the same time as it is pathologized (Goodley, 2017). “Labels such as anorexia, hysteria and agoraphobia are feminine roles enlarged to disabling conditions that blur the line between ‘normal feminine' behavior and ‘pathology” (Goodley, 2017, p. 46). Accusations of imagined illness (Wendell, 1996), malingering, drug abuse (Sheppard, 2018), or attention-seeking (Mollow, 2014; Johnson, 2015) do not target all disabled people in the same way. Nolan (2022, p. 150) reports that racialized accusations of drug abuse influence her utilization of healthcare: “Indeed, I avoid emergency rooms when my pain flares to avoid being called drug seeking because of my disability and my skin color.”

Emotions themselves can be disabling, but some disabilities tend to be dismissed as “just a feeling” (Forrest, 2020, p. 75). Vidali (2010) relates her own experience of treatment of her Irritable Bowel Syndrome to the historic case of prescribing opium to treat digestive problems that were considered to be caused by strong emotions.

“Like nineteenth century women, it seems I was being subtly prescribed anti-anxiety medication for my bowel problem, and I remember there being little conversation with my doctor regarding *why* I needed to take this drug, or what it was even supposed to do” (Vidali, 2010, n.p.).

In this way, the diagnosis of borderline personality disorder undermines the epistemic authority of persons so labeled who are cast as hypersensitive (Johnson, 2015). Similarly, the pain experience of people living with chronic pain without a diagnosis accepted in Western medicine is frequently questioned, disbelieved, and silenced—it is epistemically invalidated (Wendell, 1996; Sheppard, 2020b). Recently, the experience of long-term COVID-19 patients has been dismissed in a similar way (Bê and Sheppard, 2023).

“[M]any people with long COVID seem to have experienced the same familiar disbelief and misconceptions that are frequent for chronic conditions that are still poorly understood or easily dismissed by biomedicine” (Bê and Sheppard, 2023, p. 136).

Disabled people and their allies experience a dismissal as hysteric (in the sense of irrational) in a more figurative sense, when they express feeling hurt, rejected, or even questioned about their right to life because prospective parents terminate pregnancies as soon as prenatal testing detects genetic disorders (Shakespeare, 2011). Able-bodied mothers of disabled children are heard as making *unreasonable* demands for their disabled children in healthcare and education. They are constructed, produced, and disciplined as *mad* mothers when expressing understandable anger (Douglas et al., 2021). “[W]hen we talk of our lived experience in research, despite our feminist stance, we still fear that any claim to rigor we might wish to make will be compromised” (Douglas et al., 2021, p. 52). Saudi mothers of autistic children, more specifically, are heard as overprotective when shielding their children from feeling excluded. Teachers often accuse parents of overprotecting their disabled children—ignoring the irreplaceable impact of parental protection, involvement, and advocacy on disabled children's quality of life in excluding and discriminating societies (Sulaimani and Gut, 2020).

What does the accusation of hysteria do? Wendell (1996) shares how the idea of a body that intentionally falls ill has impacted her life. The culturally suggested notion that one has to fall sick to learn a specific life lesson triggered a potentially endless search for the root of her depression^15^, which are physically caused by her chronic illness of myalgic encephalomyelitis/chronic fatigue syndrome.

“Illness has forced me to change in ways that I am grateful for, and so, although I would joyfully accept a cure if it were offered me, I do not regret having become ill. Yet I do not believe that I became ill *because* I needed to learn what illness has taught me, nor that I will get well when I have learned everything I need to know from it. We learn from many things that do not happen to us because we need to learn from them (to regard the death of a loved one, for example, as primarily a lesson for oneself, is hideously narcissistic), and many people who could benefit from learning the same things never have the experiences that would teach them” (Wendell, 1996, p. 175).

Patsavas (2023) analyses literary representations of chronic pain that make female pain patients responsible for pain that persists. Chronic pain is explained by “improper affective attachments” (Patsavas, 2023, p. 211). One literary depiction even personifies pain as a male object of desire that seduces the female protagonist, who is cast as complicit as she chooses to *cheat* on life with isolating pain and further becomes attached to it.

When sickness is conceived as voluntary, sick people are blamed for not getting better or for becoming ill in the first place. One common allegation is that the subject shies away from working through psychological problems that underlie disease (Wendell, 1996). Nonetheless, diagnostic labels like psychosomatic disorders (Wendell, 1996; Mollow, 2014) or chronic pain (Sheppard, 2018) are often not established by diagnosing psychological problems (Wendell, 1996) but serve as a dismissal when no somatic cause can be detected (Sheppard, 2018). Although legitimate (biological) explanations in contemporary Western medicine remain limited, patients' perspectives on the cause of their symptoms are frequently unheard (Mollow, 2014). Medicalized understandings of pain, for instance, persistently demand explanations, causes, and localizations (Kolárová, 2010).

Ultimately, the accusation of intentionally falling or staying sick justifies the denial of accommodation and support, while it allows able-bodyminded people to uphold the myth of being in control over their bodies with the help of modern Western medicine (Wendell, 1996). Denied accommodations include, e.g., access to appropriate medical care (Wendell, 1996; Atkins and Hodges, 2010; Mollow, 2014; Patsavas, 2023), the scientific investigation of underlying physical processes, social or environmental causes (Wendell, 1996), or a joint responsibility for unharmful communication (Johnson, 2015).

Beyond access to treatment and medication, people living with chronic pain long for their pain to be recognized by (significant) others—an experience that they are often denied (Sheppard, 2020a). Allegations of falling or staying unwell voluntarily cast self-doubt and guilty feelings instead. Thus, patients may experience receiving a somatic diagnosis as a relief—even if being diagnosed with a profoundly disabling, progressive, or life-threatening disease (Wendell, 1996). (Non-)verbal sympathy plays a vital role in coping with and meaningfully integrating pain into one's biography (Dederich, 2020). Specifically in a period of loss and mourning, pain patients need others to validate their experience of pain and connected emotions (Sheppard, 2020a).

Medical providers play an important role in the validation of pain experiences. A paternalistic medical system casts (male) doctors as saviors who *good* (female) pain patients seek expertise from to “*teach her* about her own body” (Patsavas, 2023, p. 208; her emphasis). Challenging suggested treatment plans due to their inefficiency, side effects, or costs attached “often gets read, at best, as the very investment in pain” (Patsavas, 2023, p. 210). Moreover, doctor–patient interaction is represented as a sexualized intimate encounter, while the quest to find a doctor who doesn't dismiss pain experiences is normalized, rationalized, individualized, and minimized by a comparison to dating (Patsavas, 2023).

A third facet of medicalization manifests in epistemological disablement by the accusation of intentionally falling sick. As soon as mainstream Western medicine cannot detect a somatic cause for disease, being unwell is dismissed as just a feeling, rather than listening to the patient's perception of causes. Casting someone as hysteric comprises both pathologizing emotions as *excessive* and misunderstanding causality. In this case, sick people do not believe that their symptoms are direct effects of impairment. Therefore, medical treatment is dismissed as an option while relegating patients rather to psychological treatment.

### 3.4 Fetishizing desire

A dis/ableist culture where able-bodymindedness is *compulsory* frames any type of desire directed toward disabled bodyminds as a shameful *fetish*. Campbell (2009) comprehensively discusses four different ways to desire disability that are commonly pathologized as mad, perverse, and even unthinkable: people who desire to appear disabled (*pretenders*), people who desire to be disabled (*transabled people*), conjoint twins who desire being together and men who desire women with amputated limbs (*amputee devotees*). I further want to discuss the desire to start a family as or with a disabled person here (Hutson, 2010, 2016).

Pretenders mimic impairment or use assistive devices, while transabled people desire to have a physical or sensory impairment that matches their body image. Both ways to desire disability have been condemned by Disability Studies, which Campbell (2009) reads as revealing the community's internalized ableism.

With their ambiguous and shared boundaries of the bodymind, conjoint twins are connected to a discursive history of *monstrosity* and *freak* shows that reinscribes abjection (Campbell, 2009; Shildrick, 2010). Beyond the ambivalent constructions of conjoint twins as desirable for potential intimate partners (Campbell, 2009), their desire for conjointment has been dismissed as unthinkable. Most of the few available historical accounts state that conjoined twins were content with their interdependent way of life and desired being closely together. After all, conjoint twins share experiences up to a point where they might be indivisible into self and others (Campbell, 2009; Shildrick, 2010). Even identical twins account that they do not consider living separate lives, although living together can be challenging (Shildrick, 2010). Against the background of ableist ideals of individuality and independence, the preferability of—sometimes dangerous—surgical separation seems to unconditionally overrule any threat to the twins' emotional wellbeing, bodily integrity, and even their lives (Campbell, 2009). “[T]he question with regard to all conjoined twins is rarely if they should be separated, but rather how and how soon” (Shildrick, 2010, p. 59). Medical staff seem to think of conjoined twins as persons only when they are separated (Shildrick, 2010), while compulsory separation is justified with an ascribed increase in happiness for the disjointed twins (Campbell, 2009).

Compulsory able-bodymindedness or the normalized assumption that able-bodymindedness is always preferable casts the desire of a disabled body as a shameful fetish that needs to be hidden (Campbell, 2009; Kafer, 2012). Consequently, disabled people's intimate partners risk being misrecognized as devotees who are attracted toward (a particular) impairment and/or prosthesis (Campbell, 2009). Their denial to be a devotee is then perceived as a rejection of one's partner's disability (Kafer, 2012): “…to love an amputee is to be a devotee; to refuse such an appellation is to love an amputee only partially, ashamedly, reluctantly” (Kafer, 2012, p. 337).

Devoteeism as a specific attraction toward disability is condemned and pathologized by wider culture (Campbell, 2009). This condemnation allows able-bodyminded people to reassure themselves of their own *normal* desire and constitutes the ableist privilege that their allegedly normal desire does not need any justification. To render the desire of disabled bodies unthinkable further paves the way for disempowering, sexist and heteronormative devotee discourses. (Male) devotees capitalize on the dis/ableist assumption that they are the only people who (can) desire amputees. After all, they frame disability not only as an individual problem but centrally as a problem of female attractiveness (Kafer, 2012). Devotees use a “closed logic of desire and disgust” (Kafer, 2012, p. 338) to excuse exploitative behavior like intrusive questions, on- and offline stalking, and the distribution of amputees' personal information or photographs without consent (Kafer, 2012).

“Disabled women understand how devotee exceptionalism—‘we are the only ones who could ever love you'—perpetuates ableist assumptions about their presumed undesirability; it leaves unchallenged the notion that amputees are properly objects of disgust. Moreover, disabled women recognize the ways in which this exceptionalism is then used to excuse, if not to produce, exploitative and potentially dangerous behaviors” (Kafer, 2012, p. 342).

Not only sexually desiring a disabled partner is subject to medicalization, but also desiring family-building as or with a disabled or sick person. Disabled people are often misrecognized as asexual by their families, carers, and the wider public. When seeking medical advice in reproductive matters or to relieve sexual dysfunction, disabled people rarely perceive medical staff as supportive in finding solutions as any sexual dysfunction of impaired bodies is not framed problematic nor having children as possible (Liddiard, 2018). Hutson (2010, 2016) recounts how medical doctors dismiss her desire to have children given her chronic illness as irrational while questioning her sanity and accountability. They massively accuse her of being irresponsible and selfish in becoming pregnant as she is not making her desire to start a family dependent on technically calculated risks^16^. While able-bodiedness is chosen all the time in reproductive medicine, choosing disability is condemned as irrational, selfish, and shameful (Kafer, 2013). Parents (yet also doctors) have even been blamed for a disabled child's existence as seen in *wrongful birth* and *wrongful life* torts (Campbell, 2009). Such accusations hold particular currency for the Deaf community opposing an alleged *overcoming* of deafness via cochlear implants or when Deaf parents desire Deaf children. A Deaf lesbian couple had to endure hostility and outrage for conceiving a Deaf child with the sperm donation of a Deaf male friend (Kafer, 2013).

“Their choice to choose deafness suggests that reproductive technology can be used as more than a means to screen out alleged defects, that disability cannot ever fully disappear, that not everyone craves an ablebodied/able-minded future, that there might be a place for bodies with limited, odd, or queer movements and orientations, and that disability and queerness can indeed be desirable both in the future as well as now” (Kafer, 2013, p. 84).

What does the fetishizing of desire do? Casting any desire of disabled bodyminds as pathological evokes guilt, shame, fear, and frustration (Campbell, 2009). It delegitimizes love, attraction, and interconnectedness into feelings that should be treated or at least hidden (Campbell, 2009; Kafer, 2012). Devotee discourses block female amputees from recognizing themselves as desirable and as sexual beings beyond their amputation. They can further cut female amputees off potentially supportive communities as they induce suspicion and discomfort among (alleged) amputee women (Kafer, 2012). To fetishize the desire to have children blocks (equal) access to reproductive medicine and other support (Hutson, 2010; Kafer, 2013; Liddiard, 2018).

A medicalization of disabled people's emotions can thus manifest, fourthly, when it is delegitimized to desire disabled bodies. Intimate partners of disabled people are cast as fetishists, while the wish to start a family as or with a disabled person is dismissed as irrational.

## 4 Discussion

What does the medicalization of disabled people's emotions *do*? It allows able-bodied people to deny any complicity in the creation of unpleasant feelings that disabled people experience, e.g., feelings rooted in discrimination and exclusion. When these are framed as medical problems, medical treatment is positioned as the (only) legitimate response. Such dismissals function to individualize responsibility for disability, rather than recognizing the dis/ableist structure of society—analogous to racist, classist, or sexist structures.

The medicalization of disabled people's emotions transforms their (ascribed) feelings and emotionality in general into medical problems to be treated, yet different facets of medicalization enforce treatment in distinct ways. Normative and normalizing ideas on the *right* amount and temporality of feeling and on *normal* desire instruct disabled people and their allies to attune their feelings to hegemonic culture. Misunderstanding emotional responses to dis/ableism as *impairment effects* leads to a prescription of psychological treatment. Dismissing disabled people and their allies as *hysteric*—or should we say framing them as *mad*—undermines any entitlements or their perception as deserving of support. Both psychologizations withdraw attention elsewhere with likely adverse effects on disabled people's wellbeing, their health outcomes and healthcare delivery. The accusation of *hysteria* blocks access to medical treatment of bodily symptoms. When unpleasant emotions are mistaken as *impairment effects*, their social origin in disabling environments drops out of sight.

As I have argued elsewhere (Wechuli, 2023b), the medicalization of disabled people's emotions is relevant not only for disabled people and those offering service for them, e.g., in social work. Disabled people's emotions are medicalized in emotional discourses, which shape social relations in a dis/ableist society. When taking the dis/ableist structure of society into account, such dismissals of experience target disabled people but send a wider message about ableist performance standards and compulsory health. Moreover, one can assume that dismissals of experience might function similarly based on other markers of difference. Importantly, these dismissals are themselves raced, classed, and gendered—most obviously in the epistemological disablement of *hysteria* accusations.

The sociology of health and illness should address the medicalization of disabled people's emotions since both health and (chronic) illness are socio-cultural phenomena. Yet the dismissal of disabled people's emotions via medicalization allows able-bodyminded people to deny any complicity in causing unpleasant feelings for disabled people in a dis/ableist society. Thus, the field should challenge medicine's reach into such non-medical life areas. Moreover, as emotions are instrumentalized for medicalization the sociology of emotions should be equally concerned.

Beyond theory-building, the medicalization of disabled people's feelings is relevant to healthcare. Critically questioning the jurisdiction of medicine over disabled people's feelings promises not only more patient-centered but also more effective and fitting healthcare delivery. Equally, a key policy takeaway is to take responsibility back, e.g., from the realm of medicine, to make environments less disabling. A collectively shared responsibility for disabled people's feelings—as well as the feelings of other minoritized people—bears emancipatory potential. Noticing medicalization may foster social change toward a more inclusive and less dis/ableist society—an aspiration that does not only concern disabled people.

## Author contributions

The author confirms being the sole contributor of this work and has approved it for publication.

## Footnotes

^1^In brief, scholars of Disability Studies do not understand their central topic—disability—as epistemologically given (Boger, 2017) but rather as a socio-culturally constructed difference (Waldschmidt, 2018). In this sense, I speak of disabled people in this article to indicate being disabled by society as a social position (Waldschmidt, 2011). Although the place of chronic illness in Disability Studies remains contested (De Wolfe, 2002; Marx, 2023), sick or chronically ill people are likewise disabled in a dis/ableist society.

^2^Publications on emotional issues in the field of Disability Studies remain diverse in terms of topics, theories, and methodologies used. Given the exploratory character of the underlying study, follow-up studies will have to evaluate the coherence, fit, and points of friction more thoroughly—drawing more extensively on secondary literature.

^3^I use italics to convey that I do not find a chronology of trigger and response suitable here.

^4^Thomas (1999, 2009) understood this distinction as an analytical one—staying mindful of how it might be impossible to disentangle impairment effects and disablism in lived experiences of disability, which vary intersectionally (Thomas, 1999) and are embodied as well as socially and historically contingent (Thomas, 2009).

^5^The notion of neurotypicality points out that normal neural functioning is a construction. It is used by self-advocacy groups of people on the autistic spectrum to expose a norm that usually remains unmarked (Runswick-Cole, 2014).

^6^Although drawing conclusions about the emotional lives of autistic people who cannot compose first-hand accounts seems like an overgeneralization, the existing first-hand accounts are still deemed the best available source when speculating about other autistic people's emotional lives (Jones et al., 2001).

^7^Interestingly, schizophrenia has also been characterized as “flattened affect” (Donaldson and Prendergast, 2011, p. 130) and thus, lack of feeling, which offers a different justification for psychiatric treatment.

^8^Davis (2008) challenges the medical jurisdiction over obsession that he understands as a continuum between cultural aspiration and pathologized disorder. Some of the most common obsessive behaviors and thoughts–cleaning and checking—(only) make sense in contemporary society with knowledge on microbes and on safety hazards posed by household gadgets or city life.

^9^The genre associates childhood, able-bodymindedness, and heterosexuality with happiness (Ahmed, 2010). The plot usually combines a story where illness is being overcome with the help of Western medicine with a romance plotline that equates recovery with the heroine finding a male partner (Elman, 2012). Similarly, marriage is cast as a symbolic cure for irritable bowel syndrome and many other hystericized disorders—particularly for women (Vidali, 2010) living with chronic pain (Patsavas, 2023).

^10^Samuels (2017) does not buy into the idea that her illness, which was ultimately diagnosed as genetic, was caused by excessive grief. However, she keeps her grief about losing health, functioning, and time to herself after this incident. When grief is pathologized as melancholia, unpleasant feelings are framed as individual choice (Ahmed, 2010).

^11^Johnson (2011) reads the pathologizing of an excessive emotionality as a legacy of lobotomy, which is widely understood as an outdated medical practice yet still practiced in rare cases of, e.g., schizophrenia, despite its profoundly disabling effects.

^12^Of course, disabled people can have good reasons to underutilize medical interventions (Campbell, 2009).

^13^However, the locked-up women experience seclusion as a punitive measure and feel frightened, angry, sad, helpless, and abandoned (Fish, 2018). “Evidently, being ‘calm' does not necessarily imply a sense of well-being, it can be a manifestation of immobilization or shutting down” (Fish, 2018, p. 147). More progressive approaches to navigate anger and aggression in forensic settings recognize “that aggression occurs relationally, and that things can be done to reduce the need for seclusion, such as talking to people and acknowledging the reasons for anger and aggression” (Fish, 2018, p. 147).

^14^One professional's feelings played a paramount role in the development of the surgical practice of lobotomy. In his memoirs, lobotomy's central advocate recounts initial fear, disgust, and shame toward psychiatric patients. The sterile environment of the theater allowed him to sublimate these aversive emotions into scientific curiosity. Lobotomy was theorized retrospectively, observing changes in patients after the procedure (Johnson, 2011).

^15^An emancipatory knowledge production would trust the assessment of experts in their own right and emphasize that disease and disability are psychologically and spiritually *meaningless* here as in related scenarios (Wendell, 1996; Mollow, 2014).

^16^Hutson (2016) makes sense of her experiences as doctors repressing their own fears of vulnerability that are related to the perception of (chronic) illness as a threat rather than a part of life.
